# Bidirectional Control of Reversal in a Dual Action Task by Direct and Indirect Pathway Activation in the Dorsolateral Striatum in Mice

**DOI:** 10.3389/fnbeh.2017.00256

**Published:** 2017-12-22

**Authors:** Muriel Laurent, Jean-François De Backer, Danie Rial, Serge N. Schiffmann, Alban de Kerchove d'Exaerde

**Affiliations:** ^1^Laboratory of Neurophysiology, ULB Neuroscience Institute, Université Libre de Bruxelles (ULB), Brussels, Belgium; ^2^WELBIO, Liège, Belgium

**Keywords:** dorsolateral striatum, direct pathway, indirect pathway, learning, optogenetic activation

## Abstract

The striatum is a key brain structure involved in the processing of cognitive flexibility, which results from the balance between the flexibility demanded for novel learning of motor actions and the inflexibility required to preserve previously learned actions. In particular, the dorsolateral portion of the striatum (DLS) is engaged in the learning of action sequence. This process is temporally driven by fine adjustments in the function of the two main neuronal populations of the striatum, known as the direct pathway medium spiny neurons (dMSNs) and indirect pathway medium spiny neurons (iMSNs). Here, using optogenetics, behavioral, and electrophysiological tools, we addressed the relative role of both neuronal populations in the acquisition of a reversal dual action sequence in the DLS. While the channelrhodopsin-induced activation of dMSNs and iMSNs of the DLS did not induce changes in the learning rate of the sequence, the specific activation of the dMSNs of the DLS facilitated the acquisition of a reversal dual action sequence; the activation of iMSNs induced a significant deficit in the acquisition of the same task. Taken together our results indicate an antagonistic relationship between dMSNs and iMSNs on the acquisition of a reversal dual action sequence.

## Introduction

The ability of the brain to organize memories and action sequences in single units of cognition is of great importance for the increase in performance upon learning observed in animals. The sequence of learned actions allows the achievement of a higher efficient assessment of acquired information (Hilario et al., 2012). However, in order to achieve a higher efficient state the sequential organization of actions requires precise timing, proper initiation, and termination of the sequence (Graybiel, 1998; Yin et al., 2009; Jin and Costa, 2010).

The dorsal striatum is well-positioned to control such actions since it had been shown to play critical roles in motor planning, in the action “chunking” and in procedural learning processes (Graybiel, 1998; Yin and Knowlton, 2006; Balleine et al., 2009). Anatomically the dorsal striatum can be divided in dorsolateral (DLS) and dorsomedial (DMS) striatum. While the DLS majorly receives afferents from sensorimotor areas, the DMS receives inputs mainly from associative areas (Voorn et al., 2004; Yin, 2010). Functional data from Yin and collaborators indicate that the DMS is required for goal-directed behavior and during the acquisition of a new motor skill whereas the DLS is engaged in the automatization of a motor skill and habit learning (Yin et al., 2005; Yin and Knowlton, 2006). More specifically concerning the serial order learning, when the DMS is targeted by an excitotoxic lesion the acquisition of a simple sequence is not altered, whereas the same lesion in the DLS induces a robust decrease in the acquisition of the same task highlighting the seminal role of the DLS in the acquisition of a sequence (Yin, 2010).

The striatum is mainly composed by projection neurons (medium spiny neurons, MSNs), using GABA as their neurotransmitter. These MSNs are driven by glutamatergic cortico-thalamic inputs and are traditionally divided into two populations based on their function and neurochemical phenotype: striatonigral or striatopallidal pathways (Alexander and Crutcher, 1990; Gerfen et al., 1990; Kreitzer and Malenka, 2008); characteristically the direct MSNs (dMSNs) express dopamine D_1_ receptors (D_1_R) while the indirect MSNs (iMSNs) co-express dopamine D_2_ receptors (D_2_R) and adenosine A_2A_ receptors (A_2A_R) (Gerfen et al., 1990; Schiffmann et al., 1991). The striatal circuitry connects the direct pathway with increased locomotion and prompting actions, whereas the actions of the indirect pathway are associated with behavioral inhibition (Eagle and Baunez, 2010; Jahfari et al., 2011). However, concomitant to the development of optogenetic tools allowing the more precise assessment of the relative role of each population in different tasks has revealed that both populations act in a more coordinate and collaborative manner than previously anticipated (Cui et al., 2013; Isomura et al., 2013; Jin et al., 2014; Tecuapetla et al., 2016; Vicente et al., 2016). More specifically, it has been shown that during the initiation of learned actions both pathways are active during initiation of a sequence (Tecuapetla et al., 2016) but with distinct roles during the performance of the sequence (Jin et al., 2014). Still, the contribution of dMSNs and iMSNs to the acquisition of a reversal sequence is not clearly understood. The work of Rothwell and colleagues shown that by inactivating optogenetically dMSNs but not iMSNs the learning of a new sequence is impaired (Rothwell et al., 2015). However, the relative roles of dMSNs and iMSNs in the reversal of a dual action sequence are not known using an optogenetic activation experimental design. In contrast to previous studies our experimental approach resides on the maintenance of the tonic functioning of both, the direct and the indirect pathways during all phases of the behavioral analysis.

Here, we aimed to untangle the relative roles of dMSNs and iMSNs of the DLS, by using an optogenetic activation approach, in the acquisition of a reversal dual action sequence using a two-presses dual action operant task. Taken together, our data indicate that the specific activation of iMSNs inhibits the acquisition of a new dual action sequence, while the activation of the dMSNs facilitates the acquisition of the new sequence.

## Experimental procedures

### Experimental subjects

Male Drd1a-Cre (D_1_Cre, EY262 line) (Gong et al., 2007) or Adora2a-Cre (A_2A_Cre,) (Durieux et al., 2009) hemizygote bacterial artificial chromosome transgenic mice on a C57BL/6J background were used for behavior and optogenetic experiments. Experimental mice were 8 weeks of age and maintained on a 12-h light/dark cycle with *ad libitum* water, under controlled temperature (25 ± 2°C) and humidity. All studies were conducted in accordance with the guidelines set up by the Institutional Animal Care and Use Committee and were approved by the Ethical committee of Pôle Santé U.L.B.

### Adeno-associated virus and optic fiber stereotaxic surgeries

Mice were anesthetized with 1.5% isoflurane (Forene®, AbbVie, Wiesbaden, Germany) and underwent stereotaxic surgery to inject serotype 1 adeno-associated viruses (AAV1) (Penn Vector Core, Philadelphia, PA, USA). For behavior experiments, D_1_-Cre and A_2A_-Cre mice were stereotaxically injected bilaterally into the DLS (anterior/posterior: +0.6; lateral: ±2.3; dorsal/ventral: −3 with bregma as zero) AAV containing a double inverted open reading frame (DIO) and either ChR2(H134R)-mCherry or the control virus containing only the red fluorophore tdTomato. Virus was infused at a rate of 50 nl per minute, 0.8 μl final volume per site. The injection needle was left in place for additional 9 min following the infusion. For optogenetics, mice were implanted, bilaterally, with 3 mm chronically implantable fibers (0.37 numerical aperture, 200 micrometer core) (Thorlabs Inc., Newton, NJ, USA) using the same stereotaxic coordinates used for the viral injections.

### Instrumental training

Training took place in four operant chambers (Imetronic, Pessac, France), housed within light-resistant and sound-attenuating walls. Each chamber was equipped with a food magazine that received 14 mg Chocolate Dustless Precision Pellets® (Bio-Serv, Flemington, NJ, USA) from a pellet dispenser, with two retractable levers on either side of the magazine and house light mounted on the wall opposite the levers and magazine, with an infrared beam to record head entries into the magazine. Computers with the POLY® software (Imetronic, Pessac, France) were used to control the chambers and record the behavior.

Magazine training began with one 30-min session, during which food pellets were delivered on a random time schedule (on average every 60 s), with no levers extended, allowing the mice to learn the location of food delivery. The next day, lever-press training began on one lever (left or right, balanced between the mice). At the beginning of each session, the house light was illuminated and the lever was inserted. At the end of each session, the house light turned off and the lever retracted. Initial lever-press training consisted of 4 consecutive days of continuous reinforcement (CRF), during which the animals received a pellet for each lever press. Sessions ended at 90 min or 30 rewards (whichever came first). For sequence training, two levers L1 and L2, one on each side of the food magazine, were inserted at the beginning of each trial, which ended after two presses on any lever. The ITI was 8 s. The only reinforced sequence was L1 → L2. After 14 sessions, the reinforced order was changed to L2 → L1, and animals received an additional 14 sessions on the new sequence. During the 14 days of reversal training, the mice received blue light stimulation in the DLS during their daily session.

### Blue light stimulation

Optic fibers canula were connected to patch cord and then attached through an FC/PC adaptor to a 473 nm blue laser (DPSS 473 nm, Laserglow Technologies, Toronto, ON, Canada), and light pulses were generated through a pulse generator, Master-8 (A.M.P.I, Jerusalem, Israel). For all *in vivo* experimental protocols, 20 Hz blue light of 5 ms pulses during 20 s and a 10 s OFF interval over 90 min were delivered to all the experimental groups (D_1_-Cre and A_2A_-Cre with DLS injections of AAV-DIO-ChR2-mcherry or AAV-DIO-tdTomato). Optic fiber light intensity was measured using a light sensor (#S130A, Thorlabs Inc., Newton, NJ, USA) and light intensity ranged from 0.3 to 1 mW.

### Blue light stimulation for *c-fos* induction

Mice (D_1_-Cre and A_2A_-Cre with DLS injections of AAV-DIO-ChR2-mcherry or AAV-DIO-tdTomato) were exposed to blue light pulses for 30 min at 20 Hz. For immunohistochemistry studies mice were anesthetized, 60 min after blue light stimulation then perfused with 4% paraformaldehyde (PFA) (Sigma-Aldrich, Darmstadt, Germany). Brains were post-fixed overnight in 4% PFA then cryo-protected in 30% sucrose in PBS.

### *c-fos* immunohistaining

Striatal coronal 35 mm free-floating sections were blocked in 10% normal horse serum (NHS) and 0.3% Triton-X for 1 h before adding primary antibody. Sections were then incubated overnight at 4°C with a rabbit primary antibody: anti-*cfos* (SC52) (1/3,000, Santa Cruz Biotechnologies, Dallas, TX, USA) in PBST-1%NHS. The next day sections were rinsed in PBS then incubated in 1/200 of donkey anti-rabbit Alexa Fluor®647 (ThermoFischer Scientific, Waltham, MA, USA) in PBST-1% NHS for 1 h then subsequently rinsed in PBS.

### Patch clamp recordings

Mice were decapitated after halothane anesthesia, and coronal striatal slices (220 μm) were produced using a Vibratome® VT 1000 S (Leica, Wetzlar, Germany) in an ice-cold solution (in mM: KCl, 2.5; NaH_2_PO_4_, 1.25; NaHCO_3_, 25; MgCl_2_, 7; CaCl_2_, 0.5; Glucose, 14 and Choline chloride, 139) gassed with a carbogen solution (95% O_2_ and 5% CO_2_). The slices were then transfered to a chamber containing the aCSF solution (in mM: NaCl, 126; KCl, 2.5; NaH_2_PO_4_, 1.25; MgCl_2_, 1; CaCl_2_, 2; NaHCO_3_, 25 and Glucose, 10) at 34°C gassed with the same carbogen solution described above and allowed to rest for a minimum period of 45 min. Individual slices were then tranfered to the recording chamber continuously superfused with aCSF at a rate of 2 ml/min at room temperature under an Axioscope 2FS microscope (Carl Zeiss Instruments, Oberkochen, Germany) coupled with an iXon3 EMCCD camera (Andor Technology Ltd, Belfast, UK). ChR2-positive cells were identified using the FITC filter (525 nm wavelenght, Carl Zeiss Instruments, Oberkochen, Germany) and excited by an OptoLED® electroluminescent diode (Cairn Research Lda, Kent, England). Borosylicate pippetes (resistance between 5 and 7 MΩ) filled with in mM: KMeSO_3_, 125; KCl, 12; CaCl_2_ 0.022; MgCl_2_, 4; HEPES, 10; EGTA, 0.1; Na_2_-phosphocreatine, 5; Mg_2_-ATP, 4; and Na_2_-GTP, 0.5, were used for the recordings. Recordings were obtained with an EPC810 amplifier (HEKA Elektronik, Lambrecht, Germany) coupled with the Patchmaster system (HEKA Elektronik, Lambrecht, Germany). The spike fidelity experiments were performed in cell-attached mode (resistance > 1 GΩ) and optogenetic activation (5 ms) at a frequency of 10, 20, and 50 Hz were delivered (distributed within 1 s). The temporal synchronicity between the optogenetic activation and the occurrence of an action potential was recorded. All recordings were analyzed using the IgorPro® 6.3 software (Wavemetrics, Portland, USA).

### Statistical analysis

All data are expressed as mean ± standard error of the mean and all were submitted to Shapiro–Wilk's W normality test. Normal behavioral data was treated as a two-way ANOVA (viral injection x time as factors). Student's *t*-test against a theoretical value of 100% was employed to measure the spike fidelity; and two-tailed Student's *t*-test for independent samples for comparison of two groups (usually viral injection as seen in *c-fos* experiments). Following significant analyses of variance, multiple *post-hoc* comparisons were performed using the Newman–Keuls test. The accepted level of significance for the tests was *P* ≤ 0.05. All tests were performed using the STATISTICA software package (StatSoft Inc., Tulsa, OK, USA).

## Results

### ChR2 targeting of striatal neurons and validation of MSNs activation *in vivo*

**Figure 1:**
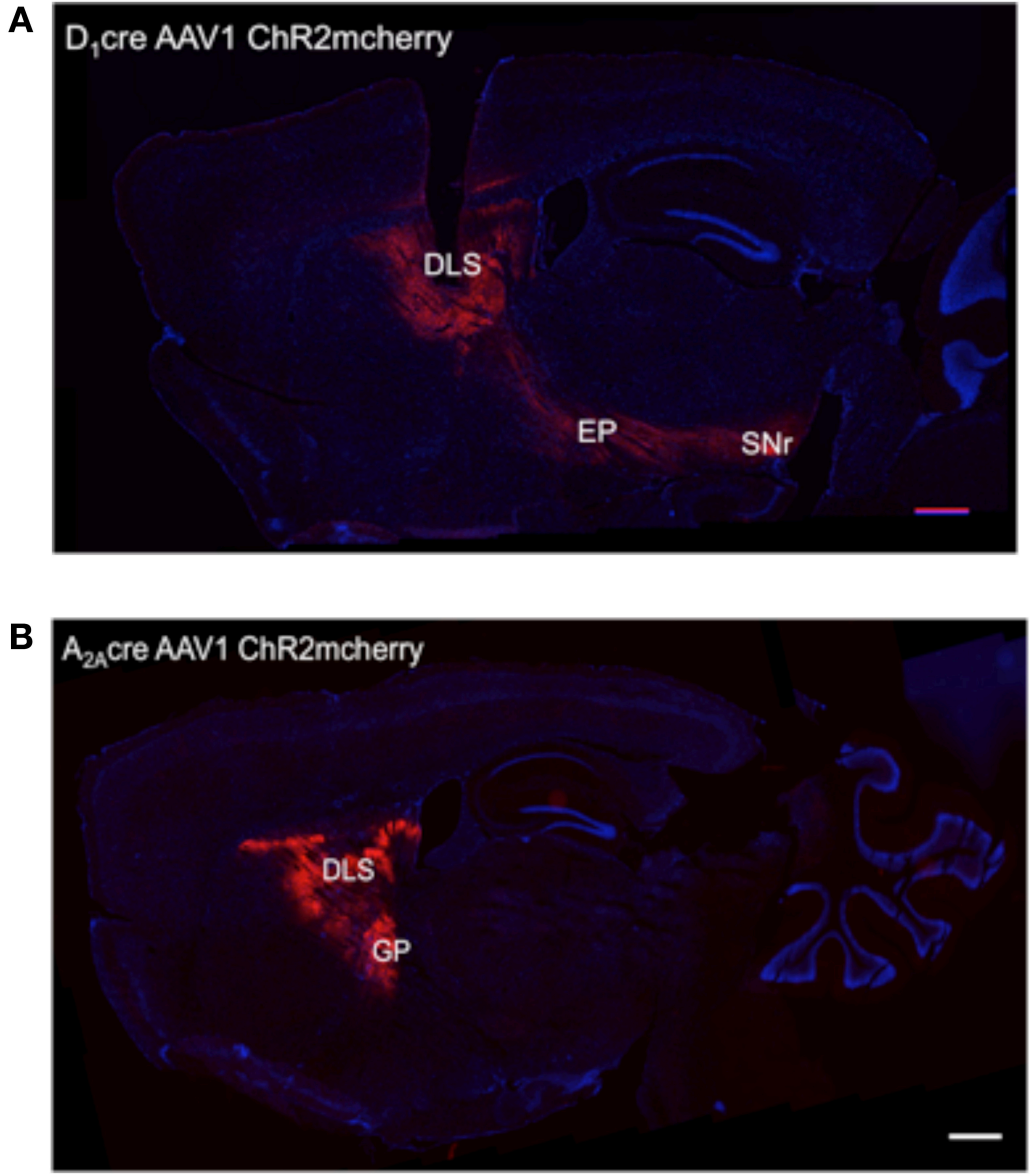
Pattern of expression of the ChR2-mCherry in DLS neurons after AAV injections. **(A)** Site of injection of AAVs into the DLS of D_1_-Cre mice and expression of ChR2 in the striatum, the entopeduncular nucleus (EP), and substantia nigra pars reticulata (SNr) indicating specificity for the striatonigral pathway. **(B)** Site of injection of AAVs into the DLS of A_2A_-Cre mice and expression of ChR2 in the striatum and the globus pallidus externus (GP) indicating specificity for the striatopallidal pathway. Scale bars are 500 μm.

To obtain selective optogenetic activation of dMSNs and iMSNs *in vivo*, we injected an adeno-associated virus (AAV1) containing a double-floxed inverted open reading frame encoding a fusion of channelrhodopsin-2 and mcherry fluorescent protein (ChR2-mcherry) into the dorsolateral striatum of D_1_-Cre (Gong et al., 2007) and A_2A_-Cre mice (Durieux et al., 2009), respectively. To confirm the expression pattern of ChR2 in the DLS, we prepared sagittal sections that include striatum, globus pallidus, and substantia nigra pars reticulata (SNr). In D_1_-Cre mice, numerous ChR2-mcherry positive fibers were observed in the striatum, traversing globus pallidus and projecting to entopedecunlar nucleus and SNr, which are the targets of dMSNs (Figure 1A). In A_2A_-Cre mice, ChR2-mcherry positive fibers were observed in the striatum projecting to the globus pallidus, but not to the entopeduncular nucleus or the SNr, consistent with proper targeting of ChR2 to iMSNs (Figure 1B).

**Figure 2:**
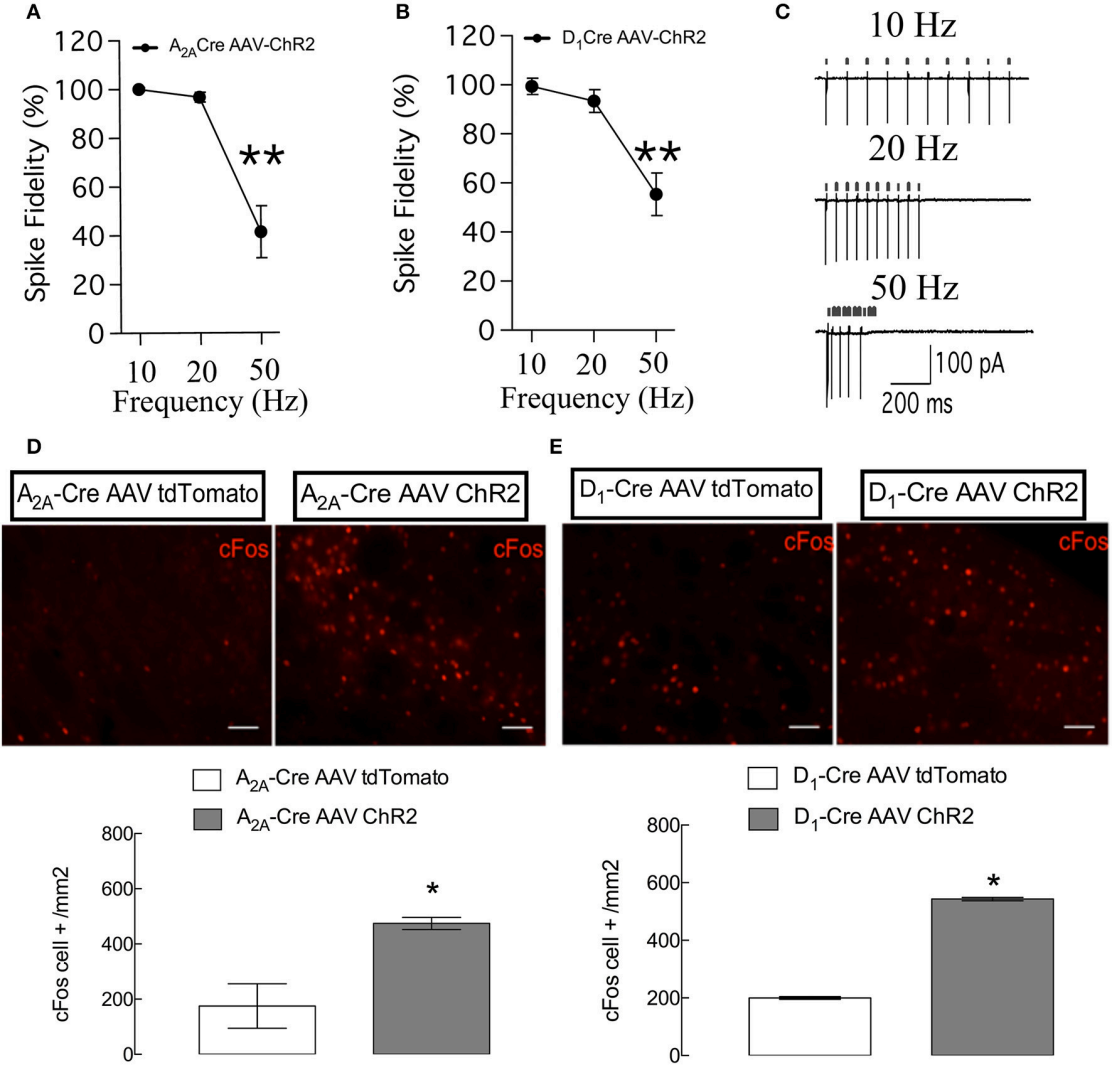
Specific control of the synchronicity between the delivery of light pulses and the presence of action potentials and *c-fos* immunostaining in the two populations of neurons of the DLS. **(A)** High spike fidelity when the frequency of 10 and 20 Hz but not 50 Hz of light is delivered in DLS slices from A_2A_-Cre **(A)** and D_1_-Cre **(B)**. **(C)** Representative recordings of 10, 20, and 50 Hz, small arrows on top of the action potentials indicate the delivery of the light pulses. Representative immunohistochemistry for *c-fos* in DLS slices from mice submitted to the behavioral tests (**D,E** top). Both populations of ChR2-expressing (iMSNs in **D** and dMSNs in **E** bottom) increased the expression of the *c-fos* gene. For spike fidelity the data are mean ± SEM of *n* = 7–8 per group. ^**^p < 0.05 when compared to the theoretical value of 100%. For *c-fos* the data are mean ± SEM of *n* = 4–5 for the A_2A_-Cre and 5–7 for the D_1_-Cre mice. ^*^p < 0.05 when compared to the respective AAV-tdTomato control. Scale bars are 50 μm.

To confirm the functionality and selectivity of ChR2, we performed cell-attached recordings in brain slices prepared from D_1_-Cre mice (Figure 2A) and A_2A_-Cre mice injected with ChR2-mcherry (Figure 2B). Illumination of ChR2-mcherry positive MSNs with 470 nm blue light delivered by trains of stimulation at a frequency of 10 and 20 Hz (*p* > 0.05, when compared to the theoretical value of 100%), but not 50 Hz (*p* < 0.05, when compared to the theoretical value of 100%), induced high spike fidelity in slices from A_2A_-Cre (Figure 2A) and D_1_-Cre mice (Figure 2B), compatible with elicited light-evoked action potentials as can be seen in the representative recordings (Figure 2C).

After the behavioral evaluation all mice received a last illumination of blue light and *c-Fos* immunolabeling was used to confirm the activation of direct and indirect pathways MSNs. Blue light illumination of MSN expressing ChR2 leads to an elevation of basal expression level of immediate early gene *c-Fos* (D_1_-Cre: *p* < 0.05, *t* = 47.36; A_2A_-Cre: *p* < 0.05, *t* = 3.59) (as can be seen in Figures 2D,E respectively) a marker for neural activity. These results confirm our ability to control MSN activity *in vivo*.

### Dual action task

**Figure 3:**
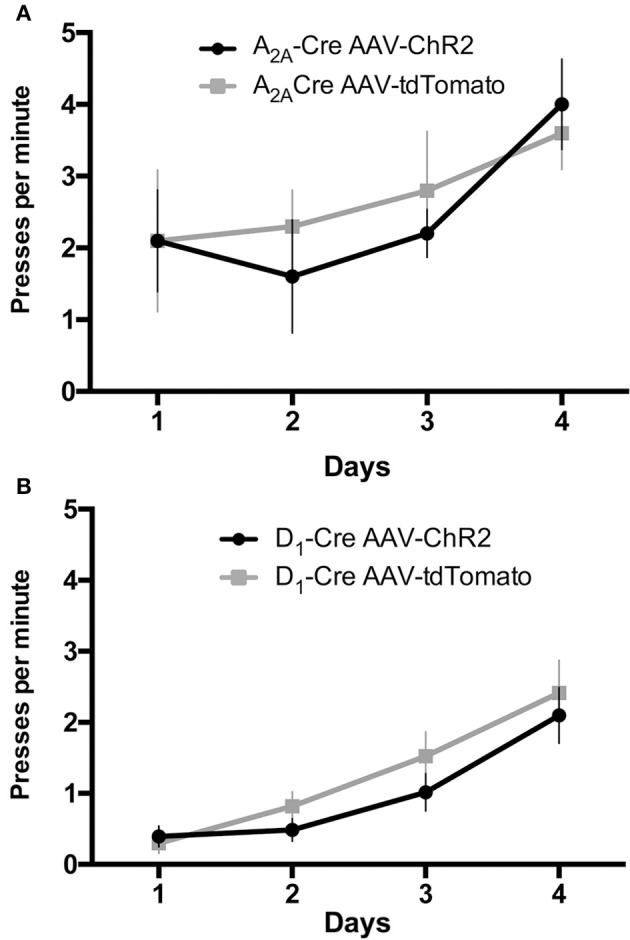
AAV injections do not affect the instrumental learning needed to perform the lever press task. During the continous reinforcement schedule the frequency of lever pressings is similar in mice injected with AAV-ChR2 or the respective controls for the iMSNs **(A)** or dMSNs **(B)**. The data are mean ± SEM of *n* = 7–12 mice per group.

**Figure 4:**
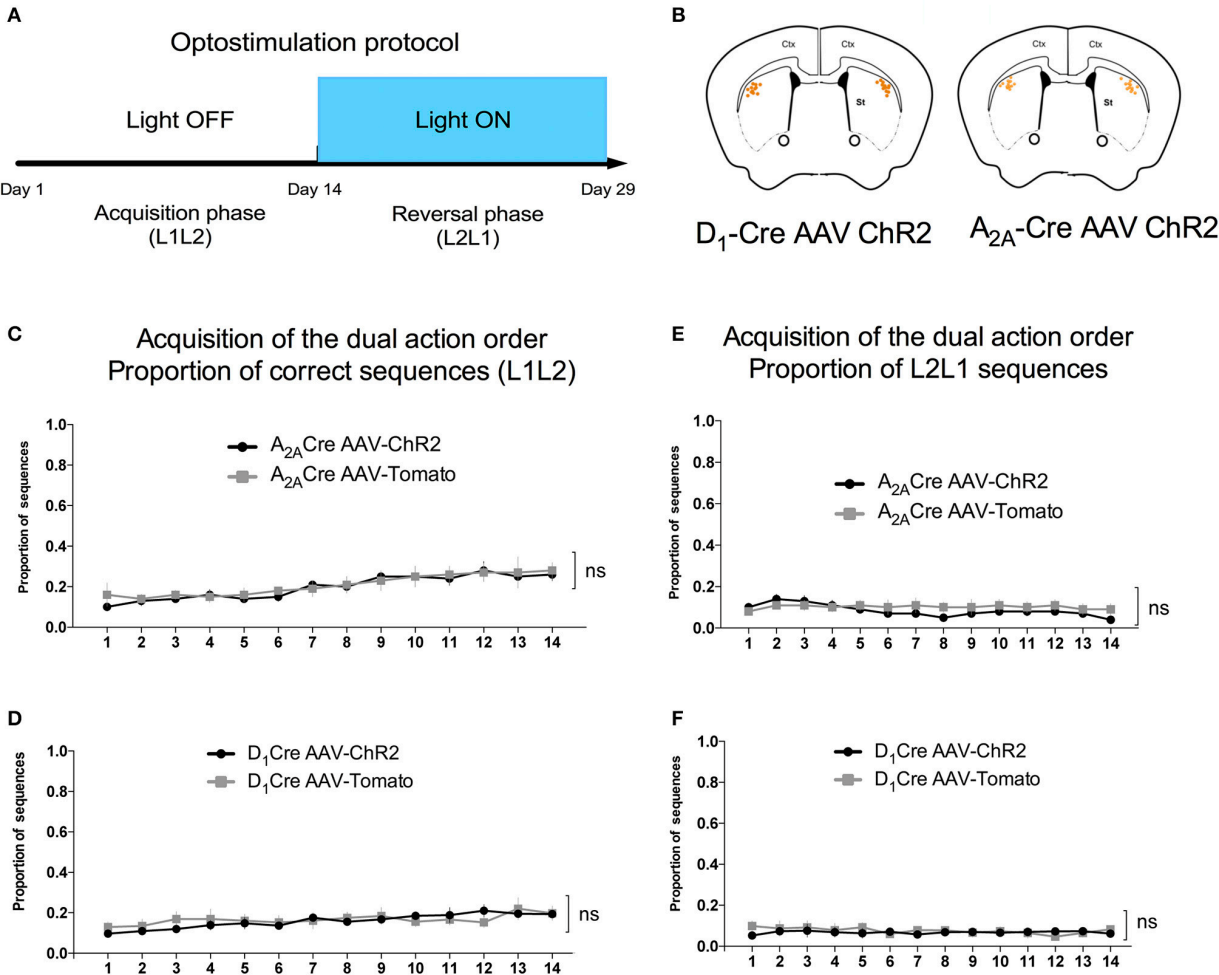
Acquisition phase of the dual action order is not influenced by viral injection. **(A)** Timeline containing the optogenetic activation protocol and **(B)** schematic figure showing the cannulaes and fiber position in the mice DLS. The proportion of correct sequences is not influenced by viral injection in both, iMSNs **(C)**, or dMSNs **(D)**. Similarly the viral injection does not affect the proportion of L2L1 incorrect sequence in iMSNs **(E)** or dMSNs **(F)**. The data are mean ± SEM of *n* = 7–12 mice per group.

D_1_-Cre and A_2A_-Cre mice injected with AAV1-DIO-ChR2-mcherry (or AAV1-DIO-tdTomato as control) and implanted with optic fiber cannulae in the DLS (AP: +0.6 mm; ML: 2.3 mm; DV: −3 mm from Bregma; positions can be visualized in the schematic **Figure 4B**) were tested for a simple self-initiated sequence task. Once inside the operant chambers the animals had to learn to execute a sequence of two presses on two different levers to receive a reward. During the first 4 days, mice were trained on one lever only to initiate the association between the action (lever press) and the outcome (receiving a reward in a food magazine). No light stimulation was delivered at this time but mice were connected to the patchcord (optic fiber connected to laser) for habituation. Figure 3 depicts the results of the acquisition of lever pressing under continuous reinforcement (CRF), in which each press is reinforced with a food pellet. All animals learned to press the lever after four sessions. AAV injections in the DLS (ChR2mcherry vs. tdTomato) had no effect on the rate of lever pressing in A_2A_-Cre and D_1_-Cre groups, as confirmed by a two-way ANOVA. For the rate of lever presses, there was a main time effect [A_2A_-Cre: *F*_(3, 68)_ = 2.76; *p* < 0.05, D_1_-Cre: *F*_(3, 68)_ = 17.35; *p* < 0.05], but no effect of AAV injections [A_2A_-Cre: *F*_(1, 68)_ = 0.19; *p* > 0.05, D_1_Cre: *F*_(1, 68)_ = 1.71; *p* > 0.05]. Thus, AAV striatal injections did not produce any significant deficit in the acquisition of the action-outcome (A-O) contingency.

### Acquisition of initial dual sequence order

After 4 days of single lever training, two levers, L1 and L2, were introduced in the chamber at the beginning of each trial, which ended after two presses on any lever. The only reinforced sequence was when animals press L1 then L2. All the other sequences were followed by extinction of the house light during 8 s with no reward delivered. Mice were trained during 14 consecutive days with one daily session.

**Figure 5:**
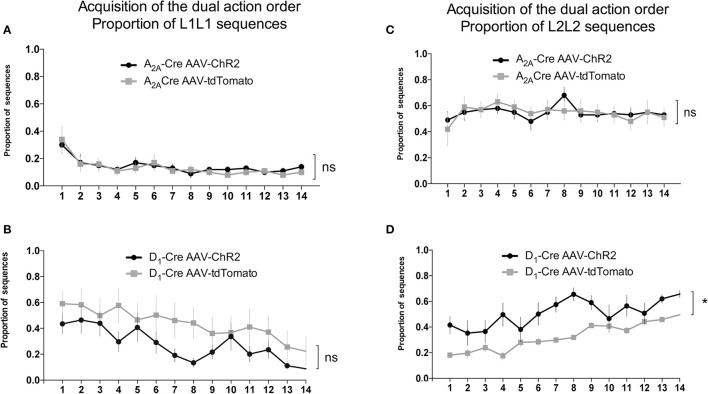
Activity of dMSNs of the DLS is involved in the chunking of the proximal to reward sequence. The decrease in the proportion of L1L1 sequence (distal to reward) is similar in both iMSNs **(A)** and dMSNs **(B)**. The proportion of the L2L2 sequence (proximal to reward) is not modified by the viral injection in iMSNs **(C)** but increases significantly in dMSNs **(D)**. The data are mean ± SEM of *n* = 7–12 mice per group. ^*^p < 0.05 when compared to the respective AAV-tdTomato control.

We used the proportion of L1L2 sequence (of all possible sequences: L2L1, L1L1, L2L2) to quantify the acquisition of the dual action order. During this initial acquisition, both groups gradually increase the proportion of correct sequence (Figures 4C,D) and this measure did not differ between groups. This observation is confirmed by a two-way ANOVA conducted on the proportion of correct sequence, with AAV injection and time as factors. There was a main effect of time [A_2A_-Cre: *F*_(13, 238)_ = 4.735, *p* < 0.05; D_1_-Cre: *F*_(13, 238)_ = 1.713; *p* < 0.05], but no effect of AAV injection [A_2A_-Cre: *F*_(1, 238)_ = 0.6502, *p* > 0.05; D_1_-Cre: *F*_(1, 238)_ = 0.5235; *p* > 0.05], and no interaction between factors [A_2A_-Cre: *F*_(13, 238)_ = 0.1896, *p* > 0.05; D_1_-Cre: *F*_(13, 238)_ = 0.5279; *p* > 0.05]. The proportion of the other sequences were also analyzed and in general no differences between the groups were found. Isolating the incorrect sequences we were not able to find differences in the proportion of L2L1 (Figures 4E,F) and L1L1 (Figures 5A,B), the proportion for these two incorrect sequences stays rather low or decreases during training. The L2L2 sequence is the most common error made by the mice, representing the repetition of the more proximal action to the reward (L2) (Figures 5C,D). While the activation of iMSNs in A_2A_-Cre mice did not induce any modification in the proportion of L2L2 error sequence [*F*_(1, 238)_ = 0.4223; *p* > 0.05] (Figure 5C), but when dMSNs in D_1_-Cre were activated the proportion of L2L2 errors increased significantly [*F*_(1, 238)_ = 4.612; *p* < 0.05].

### Reversal of the dual action order and photostimulation

14 days after the initial sequence training, the rewarded sequence was reversed to L2L1, and the mice were trained for 14 additional days. During this new training, A_2A_-Cre and D_1_-Cre mice received optogenetic stimulation (for the timeline of events see Figure 4A), activating iMSNs or dMSNs from the DLS respectively, in order to evaluate the relative role of both pathways in the learning of the new sequence.

**Figure 6:**
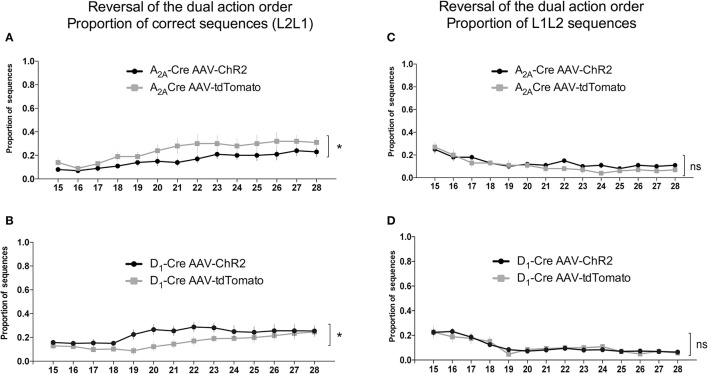
Antagonistic relationship between the indirect and direct pathway of the DLS on the proportion of correct sequences in the reversal of the dual action order. Photostimulation of iMSNs of the DLS decrease the proportion of correct sequences (L2L1) **(A)**, while when the dMSNs of the DLS are photostimulated an increase in the correct sequence is observed **(B)**. The quantification of the L1L2 (incorrect) indicate similar proportions when iMSNs **(C)** or dMSNs **(D)** are photostimulated. The data are mean ± SEM of *n* = 7–12 mice per group. ^*^p < 0.05 when compared to the respective AAV-tdTomato control.

As can be seen in Figures 6A,B when the reinforced sequence was changed to L2L1, the animals gradually learned to reverse the sequence [time factor: A_2A_-Cre: *F*_(13, 238)_ = 4.479, *p* < 0.05; and D_1_-Cre: *F*_(13, 238)_ = 2.572, *p* < 0.05]. Noteworthy is that the selective optogenetic activation of iMSNs (Figure 6A) presented a lower proportion of correct sequence compare to control group. The two-way ANOVA revealed a significant effect for the AAV injection [A_2A_-Cre: *F*_(1, 238)_ = 23.58; *p* < 0.05]. This observation indicates that activation of DLS iMSNs impairs the acquisition of a new sequence. Interestingly, when dMSNs were stimulated (Figure 6B), the ChR2 group had a higher proportion of correct sequences in comparison to controls [D_1_-Cre: *F*_(1, 238)_ = 18.37; *p* < 0.05]. However, it is important to note that after 11 days of training, the control group was able to reach the same level as the ChR2 group.

**Figure 7:**
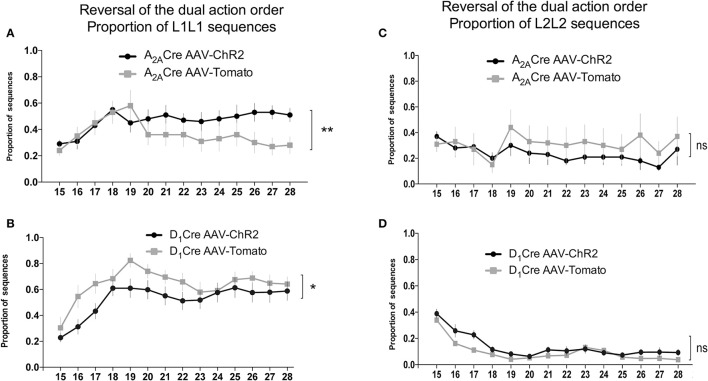
Activity of iMSNs of the DLS increases while dMSNs decreases the frequency of perseverative error of the proximal to reward sequence in the reversal of the dual action order. Optogenetic activation of DLS iMSNs **(A)** inhibit the extinction of the proximal to reward sequence (L1L1) while the optoactivation of dMSNs facilitates the extinction of the same response **(B)** without interfering with the distal to reward sequence (L2L2) for both populations **(C,D)**. The data are mean ± SEM of *n* = 7–12 mice per group. ^*^p < 0.05, ^**^p < 0.01 when compared to the respective AAV-tdTomato control.

The proportion of the others sequences were also analyzed. The evaluation of the proportion of L1L2 (Figures 6C,D) and L2L2 (Figures 7C,D) sequences revealed similar responses between the ChR2 and the control group. However, the analysis of the proportion of L1L1 (repetition of the proximal action) revealed a biphasic response from the A_2A_-Cre control group (high proportion at the beginning but decreasing over days), while the proportion of the same incorrect response remained high [time factor: A_2A_-Cre: *F*_(13, 238)_ = 1.668; *p* > 0.05] after the optogenetic activation of the iMSNs (Figure 7A) [A_2A_-Cre: *F*_(1, 238)_ = 13.41; *p* < 0.05]; [interaction: A_2A_-Cre: *F*_(13, 238)_ = 1.180; *p* > 0.05]. The same analysis for the D_1_-Cre mice revealed that L1L1 is also the most frequent incorrect sequence (Figure 7B). However, the frequency of L1L1 errors of the ChR2 group was significantly lower in comparison to the respective control as revealed by the two-way ANOVA [D_1_-Cre: *F*_(1, 238)_ = 19.10; *p* < 0.05], time [D_1_-Cre: *F*_(13, 238)_ = 5.226; *p* < 0.05], but no interaction between those factors [D_1_-Cre: *F*_(13, 238)_ = 0.4792; *p* > 0.05] (Figure 7B).

## Discussion

We examined the effects of the selective optogenetic activation of iMSNs and dMSNs of the DLS on the acquisition of a simple sequence. The injection of AAV_1_ did not *per se* impair the acquisition of a simple sequence, however, a significant deficit in the acquisition of a new sequence was observed when the indirect pathway was optogenetically stimulated. In contrast, the activation of the direct pathway improved the acquisition of the same sequence. These data reveal a critical differential role of dMSNs and iMSNs in the acquisition of a dual action order in mice.

It had been shown previously that excitotoxic lesions of all striatal neurons of the DLS disrupt the dual action order learning (Yin, 2010). Combining this information with the results here gathered, it is suggested that at least in physiological conditions, the role of dMSNs is hierarchically predominant over the iMSNs to initiate learning. Additionally, we observed that the most frequent error made by the mice was due to the repetition of the lever pressed (L2L2 and L1L1). In control animals the L1L1 error decreases over repetition. This pattern of errors suggests that, at first in the acquisition of a sequence, the proximal action is initially favored and the presence or not of the reinforcement will select the closest operant action leading to the reward. Only after repeating incorrect sequences leading to no reward, the animal will gradually distinguish and switch between the two sequences to favor the one resulting in reward. However, when iMSNs were activated, the repetition of the proximal action (L1L1 error) remained frequent at a random proportion with different types of errors, namely L2L2 and L2L1, eliminating the possibility of a general inability to discriminate between different sequences. The same rationale is applied to the possibility of a perseverative choice because the proportion of L2L2 sequence gradually reduced over time. Thus, this pattern of error suggests that activation of iMSNs in A_2A_-Cre mice leads to a selective deficit in the connection between different actions to form a correct memory. It is plausible that the optogenetic activation of iMSNs neurons leads to a disparity in the striatal output, causing a deficit in the sequence completion. On the other hand the activation of dMSNs increased the frequency of the L2L2 error, suggesting that in the DLS the direct pathway contributes actively in the chunking of the distal part of the information.

According to the classical view of basal ganglia function, dMSNs are part of a “go” pathway that facilitates movement while iMSNs are part of a “no go” pathway that suppresses undesired movements (Albin et al., 1989). While studies using genetic inactivation and optogenetic stimulation confirmed the classical antagonistic view of the striatonigral and striatopallidal neurons (Kravitz et al., 2010, 2012; Freeze et al., 2013), others suggest a more cooperative and at all points more complex interaction between the two neuronal populations (Cui et al., 2013; Jin et al., 2014). Sippy and colleagues provided evidence on the possible dynamics of this interaction, after dMSNs encoded the “go” signal to initiate action, both subpopulations (dMSNs and iMSNs) behaved similarly (Sippy et al., 2015). This suggests validity for both views since the engaging of a “go” or “no-go” signal depends on the subtype of neurons prevailing, but after that, the subpopulations interact in a cooperative way. In agreement with this view, during habit formation, dMSNs fire before iMSNs indicating that this timing imbalance in activation correlates with action initiation, but for the completion of a correct sequence both systems need to act in a balanced and synchronous manner (O'Hare et al., 2016). It is worth mentioning that one limitation of our results is the presence of the collateral inhibition between the direct and the indirect pathway that may play a role in our dual action task, masking the selectivity of the activation of direct or indirect pathways (for review see Burke et al., 2017).

Still, considering the complexity of the task here presented it is challenging to picture the lack of participation of cortical projections in the reversal learning of a dual order sequence, especially taking in account that the genetic deletion of NMDA receptors connecting to striatal MSNs is sufficient to decrease the rate of learning of certain sequence suggesting that striatal plasticity is necessary for appropriate organization of sequential actions (Jin and Costa, 2010; Jin et al., 2014). Moreover, Rothwell et al. showed that excitatory synapses connecting the secondary motor cortex (M2) to dMSNs in the DLS regulate the performance of a dual action order task (Rothwell et al., 2015). In perspective, deciphering the participation of the corticostriatal synapses in the reversal learning of a dual action sequence remains to be addressed.

## Author contributions

ML and AKE: designed the work; ML: performed the optogenetic, behavioral, and neurochemical experiments; ML, SS, and AKE: analyzed and interpreted the results; J-FD and DR: performed, analyzed, and interpreted the results from electrophysiological recordings; ML, DR, and AKE: wrote the paper; AKE: supervised all aspects of the work. All authors discussed findings, edited, and contributed to the final version of the manuscript. All authors approved the final version of manuscript.

### Conflict of interest statement

The authors declare that the research was conducted in the absence of any commercial or financial relationships that could be construed as a potential conflict of interest.
